# Nano and Microsensors for Mammalian Cell Studies

**DOI:** 10.3390/mi9090439

**Published:** 2018-08-31

**Authors:** Ioana Voiculescu, Masaya Toda, Naoki Inomata, Takahito Ono, Fang Li

**Affiliations:** 1 Mechanical Engineering Department, City College of New York, New York, NY 10031, USA; 2 Graduate School of Engineering, Tohoku University, Sendai 980-8579, Japan; inomata@nme.mech.tohoku.ac.jp (N.I.); ono@nme.mech.tohoku.ac.jp (T.O.); 3 Mechanical Engineering, New York Institute of Technology, New York, NY 11568, USA; fli08@nyit.edu

**Keywords:** cantilever beam, resonant frequency, impedance spectroscopy, electric cell-substrate impedance sensing

## Abstract

This review presents several sensors with dimensions at the nano- and micro-scale used for biological applications. Two types of cantilever beams employed as highly sensitive temperature sensors with biological applications will be presented. One type of cantilever beam is fabricated from composite materials and is operated in the deflection mode. In order to achieve the high sensitivity required for detection of heat generated by a single mammalian cell, the cantilever beam temperature sensor presented in this review was microprocessed with a length at the microscale and a thickness in the nanoscale dimension. The second type of cantilever beam presented in this review was operated in the resonant frequency regime. The working principle of the vibrating cantilever beam temperature sensor is based on shifts in resonant frequency in response to temperature variations generated by mammalian cells. Besides the cantilever beam biosensors, two biosensors based on the electric cell-substrate impedance sensing (ECIS) used to monitor mammalian cells attachment and viability will be presented in this review. These ECIS sensors have dimensions at the microscale, with the gold films used for electrodes having thickness at the nanoscale. These micro/nano biosensors and their mammalian cell applications presented in the review demonstrates the diversity of the biosensor technology and applications.

## 1. Introduction

This review presents two types of biosensors with dimensions at the nano- and micro-scale used for biological applications focused on live mammalian cells. The biosensors in this review are: the cantilever beam and electrochemical sensor based on electric cell-substrate impedance sensing (ECIS) technique. The cantilever beams presented in this review are used as temperature sensors and are operated in deflection mode or in resonant frequency mode. The ECIS technique is a label-free, real-time, and noninvasive method to monitor the cell attachment, viability and proliferation and the cellular response to mechanical stimuli. In this review the ECIS technique is used to study the mammalian cells viability.

**Figure 1:**
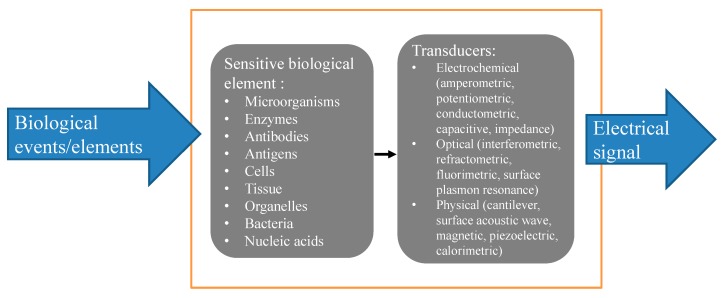
Principle of biosensors.

A biosensor is an analytical device, used for the detection of an analyte, that combines a sensitive biological component with physicochemical detector [1,2,3]. The sensitive biological element could be a biomimetic component or biologically derived material that interacts or binds the analyte under study. A wide range of biological material is used for the biosensors such as tissue, microorganisms, organelles, mammalian cells, bacteria, enzymes, antibodies, nucleic acids, etc. [3,4,5,6,7,8,9,10,11,12,13,14,15]. The detector element (transducer), which transforms the biological signal into a physicochemical signal, has different transduction options: electrochemiluminescence [16,17,18,19], optical [20,21], electrochemical [22,23], fluorescence [24,25], piezoelectric [26], etc., resulting from the interaction of the analyte with the sensitive biological element. Figure 1 represents an illustration of the principle of biosensors. 

The biosensor dimensions are in the micro- and nano-scale [1,2,27] so the fabrication technologies can be MEMS, CMOS, and nanotechnology [27,28,29]. The current trend for biosensors is integration in point of care (POC) [30,31,32]; wearable sensors are also used [33,34]. Point-of-care tests are simple medical tests that can be performed at the bedside. POC devices are simple, portable, and present the results on cell phones or computer displays. Wearable Biosensors (WBS) are gaining interest nowadays, and currently, they promise to be an important development in the sector of wearable health technology. Generally, WBS are used for healthcare applications related to sport and the military. The use of wearable monitoring devices or wearable biosensors that allow constant monitoring of physiological signals is essential for the advancement of both the diagnosis and treatment of diseases. Wearable biosensor systems are devices that allow physicians to monitor individuals over weeks or months. This novel technology typically relies on wireless sensors enclosed in bandages or patches, or in items that can be worn. 

In past ten years, stretchable electronics have been widely investigated. Dr. John A. Rogers’ group in the University of Illinois at Urbana-Champaign invented the stretchable electronics concept [35]. They developed several stretchable sensors for monitoring the heart such as: pH sensors, temperature sensors, (electrocardiogram) ECG sensors, and a microscale, inorganic light-emitting diodes stretchable sensor [36,37,38,39,40]. These stretchable sensors were fabricated on the same membrane, and provided conformal interfaces to all points on the heart enabled by the soft elasticity of the membrane itself [37]. A skin-mounted, non-invasive sensor is another application of stretchable electronics. The metallic part of the sensor is fabricated from gold of a nanometer thickness that is patterned into a network of serpentine ribbons for reduced stiffness and enhanced stretchability. These stretchable sensors allow electrophysiological signals measurements with ultrathin, skin-like polymer sheets that conformally laminate onto the surface of the skin in a manner that is mechanically invisible to the user, much like a temporary transfer tattoo [38,39,40].

Recently, several studies have been reported on stretchable electronics integrated into cell culture system or organ on chip systems for cell monitoring. A stretchable membrane with micro electrode arrays (MEA) was used to record the electrical activity of human pluripotent stem cell-derived cardiomyocytes (hPSC-CMs) plated on the stretchable membrane [41,42]. In the literature, a stretchable electrode to perform cyclic voltammetry and detect the nitric oxide produced by the mammalian cells when stretched was also reported [43]. This technique requires an electrolyte which may affect the mammalian cell integrity, and cannot give information about the cell morphology. Another stretchable electrode was developed through the formation of wrinkles on the surface of a 90 nm thick conductive layer of poly(3,4-hylenedioxythiophene):poly(styrene sulfonate) (PEDOT:PSS) on a pre-stretched 130 μm thick poly(dimethylsiloxane) (PDMS) substrate. The electrode was demonstrated to combine good stretchability, conductivity, biocompatibility, and optical transparency. Through the measurement of impedance between two ends of a single electrode, they demonstrate the capability of conductive substrates to measure changes in ionic transmembrane currents due to mechanical stretching [44].

This paper reviews the research of the authors in the area of biosensors for mammalian cell applications. The micro electromechanical systems (MEMS) cantilever beam is an important structure that has been demonstrated as a sensitive and reliable thermomechanical sensor for chemical and biological detections [45,46,47,48,49]. The presented cantilever beams are fabricated from dissimilar materials to explore the effect of different coefficients of thermal expansions of the materials. The cantilever beam operated in the deflection mode is a bimaterial cantilever beam fabricated from silicon nitride and thin metallic films as gold or aluminum. When the temperature varied, the different values of the coefficient of thermal expansion of metal and silicon caused the sensor to deflect. Extremely small variations of temperature produce a differential stress due to the thermal bimorph effect, and generate the cantilever beam deflection at its free end. The heat could be applied at the end of the bimaterial cantilever beam temperature sensor, or uniformly along the entire length of the sensor. In order to achieve the high sensitivity required for the detection of heat generated by a single mammalian cell or very small number of cells, the cantilever beam temperature sensor presented in this review was microprocessed with the length at microscale and the thickness at nanoscale dimensions [50,51,52,53].

The second type of cantilever beam presented in this review was operated in the resonant frequency regime [54,55]. The vibration property of cantilever beams relates the temperature change to the Young’s modulus. In the view of thermal sensing, higher dependence of temperature changes is more reliable. The resonant cantilever beam was also demonstrated as a sensitive temperature sensor for biological applications. As a thermogenic sample, the brown fat cells (BFCs) that are related with metabolic heat production were employed [54]. The working principle of the resonator cantilever beam temperature sensor is based on shifts in resonant frequency in response to temperature changes. The measurements were performed by stimulating the activity of BFC by flowing norepinephrine (NE) solution (1 μmol/L) [50].

The concept of the cantilever beam calorimeter was pioneered by Gimzewski and Gerber, who, for the first time, transformed an atomic force microscope (AFM) cantilever beam into a calorimeter by coating the AFM silicon nitride cantilever beam with a thin film of aluminum [56,57]. The bimaterial cantilever beam calorimeter has various applications in diverse areas. The variation of heat flux in cantilever beam can be caused either by external influences or actions occurring directly on the cantilever beam surface by catalytic reactions. For the detection of combustible materials and explosives [58], the calorimeter includes a microheater that generates the auto-ignition of the combustible vapors. The ignition occurs at different temperatures and it is monitored by the variations of the cantilever beam resonant frequencies or bending. Another important application of the cantilever beam calorimeter is as infrared (IR) detector [59,60,61,62,63,64,65,66]. In this situation the cantilever beam bends in response to the heat produced by IR illuminations. The cantilever beam calorimeter could also detect chemical or biological analytes. The presence of the chemical or biological species is detected due to the infared (IR) absorption by the biological analyte that generates heat [67,68,69]. 

Besides the cantilever beam temperature sensors, two biosensors based on ECIS technique will also be introduced in this review. The ECIS technique has several important applications, such as monitoring cell migration and wound repair [70]. The ECIS technique is successfully used for monitoring cell proliferation [71]. As cells proliferate, two factors act to change the impedance: cell number and morphology. In most instances, the cells grow asynchronously and the impedance gradually increases until a maximum when cells become confluent. The impedance change is approximately linear with cell number while the cells are forming a monolayer. ECIS provides the highly sensitive, real-time continuous trans epithelial electrical resistance (TEER) measurement required for barrier function studies [72]. ECIS is especially useful to monitor the signal transduction pathways activated by G protein coupled receptors (GPCR). GPCR activation, regardless of the second messenger, results in alterations of the cell’s cytoskeletal elements, causing morphological changes that can be monitored with the ECIS technique [73]. When cells differentiate, they change their behavior, allowing the ECIS technique to follow the events of cell differentiation [74]. The ECIS system has been used specifically to assess the cytotoxicity of a variety of toxins. ECIS-based toxicity tests are far superior to simple cell death assays, because cell function is also monitored [75]. The ECIS technique is used for monitoring cell attachment and spreading. Traditional counting attached cells assays can only quantify the number of cells attached to any ECM coating. ECIS assays give feedback on the strength of the attachment of the cells to the extracellular matrix (ECM) [76]. ECIS can distinguish between transmigration mechanisms that leave the monolayer intact from those that disrupt the cell layer. Published examples include metastatic cell and leukocyte transendothelial migration, as well as the migration of pathogens such as yeast, anthrax, streptococcus, plasmodium, trypanosomes, and spirochetes [77]. ECIS technique can also monitor cell inflammation. In this respect, ECIS recovery-after-wounding assays allow for the discovery of molecules which aid in the process of tissue repair. ECIS barrier function assays specifically measure the response of epithelial and endothelial cells to secreted cytokines, and can give indirect information about the binding of immune cells to the epithelium or endothelium [78]. ECIS has important applications for the study of cancer metastasis. The study of cancer metastasis incorporates many of the applications above in ways that reflect the specific biology of cancer [79]. 

One of the sensors discussed in this review is a quartz crystal microbalance (QCM) resonator that is integrated with the ECIS technique in a single device [80,81]. This sensor contributes to increasing the security of drinking water, and was used to assess water toxicity. The second ECIS sensor is based on flexible electronics technology [82]. This is the first time when ECIS electrodes were fabricated on a stretchable polydimethylsiloxane (PDMS) substrate and ECIS measurements on mammalian cells exposed to cyclic strain were performed. The stretchable ECIS biosensors simulate in vitro the dynamic environment of organism, such as pulsation, bending and elongation/contraction, which enables investigations on cell behavior that undergoes mechanical stimuli in biological tissue. Bovine aortic endothelial cells (BAECs) have been used as culturing mammalian cells for the stretchable ECIS technique, because the BAECs are exposed in vivo to cyclic physiologic elongation produced by the blood circulation in the arteries. This innovative stretchable ECIS sensor has the ability to analyze cell proliferation, determine cell number and density, and apply mechanical stimulation at the same time.

## 2. Nano and Micro Cantilever Beam Temperature Sensor for Biological Applications 

### 2.1. Principle of Operation of the Cantilever Beam Temperature Sensor 

**Figure 2:**
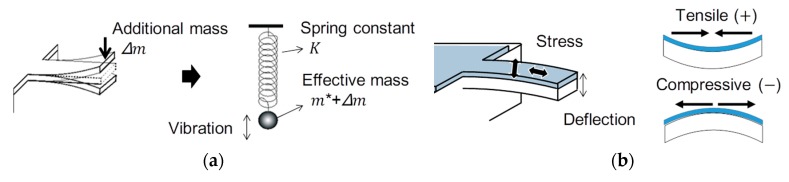
Principles of operation of the cantilever beam. (**a**) Equivalent spring and mass concept for simulation of vibrations of cantilever beam; (**b**) The bending mode of cantilever beam due to the stress on the surface, adopted from [83].

The cantilever beam sensor typically has two operating modes. One is the vibration mode, which measures resonant frequency changes due to additional mass at the apex of the cantilever beam. For the vibration mode, the cantilever beam is considered as a simple spring with an effective mass connected to the free end (Figure 2a) [83]. The second operation mode of the cantilever beam is the bending mode, which measures the static deflection of the cantilever beam. This mode is very useful especially in biotechnology applications when biological material is connected on the cantilever beam. The deposited film on cantilever generates the surface stresses for bending upward (tensile stress, +) or bending downward (compressive stress, −) due to the swelling or shrinkage effect as the response to adsorption of biological materials (Figure 2b) [83].

### 2.2. Bimaterial Cantilever Beam Temperature Sensor

The bimaterial cantilever beam temperature sensor presented in this review has an innovative structure fabricated from thin layers of silicon nitride and gold. In order for the cantilever beam to generate a large deflection in the presence of very small temperature variations, the bimaterial beam was conceived to be long and extremely thin, with a length in the range of micrometers and a thickness in the range of nanometers. The fabrication of a long cantilever beam with a length equal to hundreds of micrometers and a thickness at nanoscale dimensions is difficult, and the calibration of this cantilever beam when employed in the liquid is challenging. To our knowledge, no similar cantilever beam temperature sensor for biological applications has been reported in the literature. 

The bimaterial microcantilever beam is fabricated from two dissimilar materials with different coefficients of thermal expansion. Due to the different values of the thermal expansion coefficients, the bimorph cantilever beam deflects in response to very small temperature variations, and could be employed as a sensitive calorimeter.

The bimaterial cantilever beam presented in this review was designed to be used for chemical and biological applications. The cantilever beam was fabricated from a low stress SiNx film (0.9 < x < 1) as the main structure, and was covered with an Au layer. This cantilever beam was operated in the bending mode, and therefore, its length is in the micrometer range and its thickness in the nanometer range. 

The fabrication started from a silicon wafer with a thickness of 300 μm, and is presented in literature [50,51,52,53,54].

**Figure 3:**
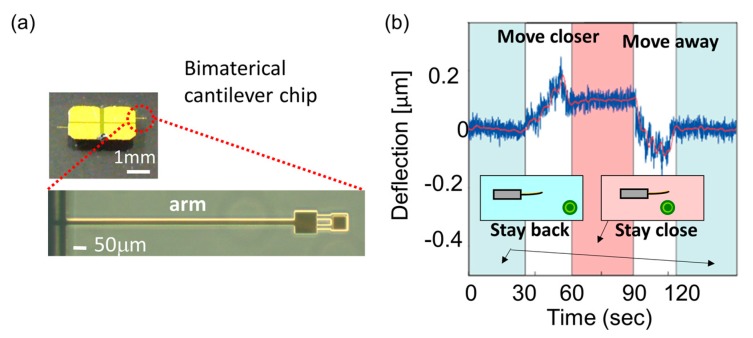
(**a**) Optical images of the completed chip with cantilever sensors; (**b**) Bimaterial microcantilever displacement versus time. Temperature change of the BFCs after adding NE; with the BFCs staying close to the sensor or the BFCs moving away, adopted from [83].

The dimensions of the cantilever beam were 750 × 20 µm, length and width respectively. The cantilever beam composite structure was gold (100 nm in thickness) and silicon nitride (200 nm in thickness). Images of the completed chip with cantilever sensor are shown in Figure 3a. The cantilever beam deflection was measured under a conventional microscope, when 6 cells of BFCs were situated about 5 µm from the tip (Figure 3b) after the BFCs thermal stimulation with NE. When the cantilever beam senses the heat, it bends due to the difference of the coefficient of thermal expansion of the composite structure. In the absence of the local heat source, there is no noticeable displacement of the microcantilever tip, whether the cells are close to it or not, (~400 μm apart), although the microcantilever is disturbed briefly, as the BFCs are flowing in the beam vicinity. In contrast, the tip was displaced from its original position when the same manipulations were performed after NE was added, and the displacement increased over time. The displacement of the microcantilever tip continued to increase for a few hours before starting to decrease. After 60 min from the addition of NE, the displacement of cantilever of the maximum value (98.4 nm) was observed. The heat response of the cells was clearly observed by the bimaterial cantilever beam sensor; however, the heat response of the cells seems to be individual, with different responses based on each cell. 

The bimaterial cantilever of 500 × 20 μm^2^ was experimentally calibrated in water using a platinum/rhodium wire micro heater with a length of 20 μm and a diameter of 5 μm. Details are provided in the supporting material 2 of the reported paper [50]. The displacement of the bimaterial cantilever when the artificial local heat source was applied to the apex was recorded. The 254 nm/K of temperature sensitivity was determined. This type of cantilever beam is sensitive to temperature variations produced by single cells.

### 2.3. Resonant Cantilever Beam Temperature Sensor

Second type of cantilever beam of this review is operated in a resonant frequency regime. This resonant cantilever beam was fabricated using conventional microfabrication techniques such as photolithography, deposition, etching, and anodic bonding. The resonant cantilever beam was fabricated from Si and sandwiched between two glass slices to create two small enclosed spaces; one was a microfluidic channel that contains part of the cantilever beam and the BFCs and media, and the other enclosed space was a microvacuum chamber that contains the resonant cantilever beam temperature sensor. One Tempax glass slice was employed for the top of the device, and the other for its bottom. They were patterned by hydrogen fluoride (HF) etching. For Si cantilever beam part silicon-on-insulator (SOI) was used. The fabrication of this cantilever beam biosensor is presented in the literature [55]

**Figure 4:**
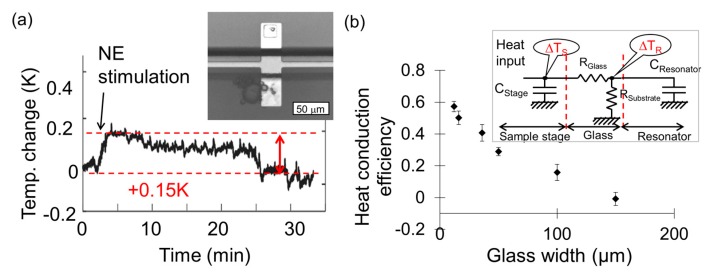
(**a**) Optical micrograph of the fabricated device and the sketch of the cross-section; (**b**) Temperature change of the BFCs after adding NE. The inset shows the BFCs attached to the sample stage, adopted from [83].

The resonant cantilevered beam dimensions were 30 μm in width, 50 μm in length, and 1.5 μm thickness. The microchannel was 20 μm in depth and 100 μm in width. In the double-supported resonator device, the resonator dimensions were 30 μm in width, 75 μm in length, and 0.4 μm in thickness. The sample stage was 30 μm in width, 50 μm in length, and 0.4 μm in thickness. The microchannel was 20 μm in depth and 50 μm in width. Figure 4a shows an optical microscope image of the fabricated device. Figure 4b illustrates the temperature change of the BFCs after adding NE. 

The heat sensing of a single cell is demonstrated using this resonant cantilever beam. The working principle of the resonator is based on shifts in resonant frequency in response to temperature changes. As a thermogenic sample, BFCs that are related with metabolic heat production were employed. This cantilever beam was supported in the median region, so both ends of this type of beam were free. One end of the cantilever beam resonator was enclosed in the micromachined microchamber where a vacuum was created. The opposite side of the resonant cantilever beam was immersed in the microfluidic channel used for BFC culture. The resonant frequency and the temperature coefficient were 960 kHz and 22.0 ppm/K, respectively. The temperature measurements were performed by stimulating the activity of BFC by introducing NE solution (1 μmol/L) in the microfluidic chamber that contains BFC culture. The cells were introduced in the microfluidic channel, and some cells spontaneously attached to the sample stage side of the cantilever beam, Figure 4a. Then resonant frequency measurements of the cantilever beam side contained in the vacuum were performed. Heat production was observed for a few minutes after adding NE, and the heat generation continued for approximately 23 min, as shown in Figure 4b. BFCs began to consume oxygen and generate heat a few minutes after adding the NE as stimulus. A temperature change of 0.27 °C corresponds to 1 nJ (nano Joule) of heat, which is the energy of proton gradient in mitochondrial inner membrane dissipated as heat. The experimental result of the single BFC corresponds to measurements in performed on cells aggregates. This device is useful for observing the thermal phenomenon of biological cells [55].

## 3. Electric Cell-Substrate Impedance Sensing (ECIS) Sensor for Mammalian Cells Applications

### 3.1. Electric Cell-Substrate Impedance Sensing (ECIS) Technique

ECIS is a real-time and label-free detection method to analyze the behavior of cells. The ECIS technique was pioneered by Giaever and Keese [84,85,86], and has been extensively studied for over two decades due to its simple structure, easy operation, and sensitivity to many cell behaviors and properties [84,85,86,87,88,89,90,91,92,93,94,95,96]. The ECIS technique can measure cell attachment, proliferation, migration, invasion, and cell viability because the impedance measurements are direct responding to cell attachment, growth, and proliferation. When cells attach onto the electrodes, cell attachments result in additional impedance to the circuit. Their insulating properties can be detected. The impedance values gradually increase until monolayer formation, before reaching an equilibrium when the cells are confluent and stable. Apoptotic cells lose the dielectric properties and tend to detach from the sensing electrodes. When the cells detach or lose their dielectric properties, the measured membrane impedance will decrease. As cells grow and cover the electrodes, information about the morphology of the cells and nature of the cell attachment can be extracted from the measured impedance [86,89,90].

**Figure 5:**
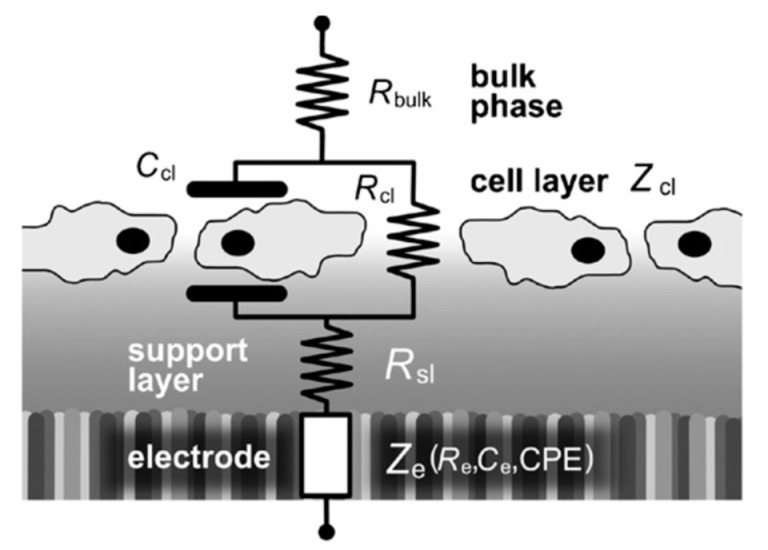
Experimental design of mammalian cells on fibronectin layer along with corresponding equivalent circuit characteristic for impedance spectroscopy.

**Figure 6:**
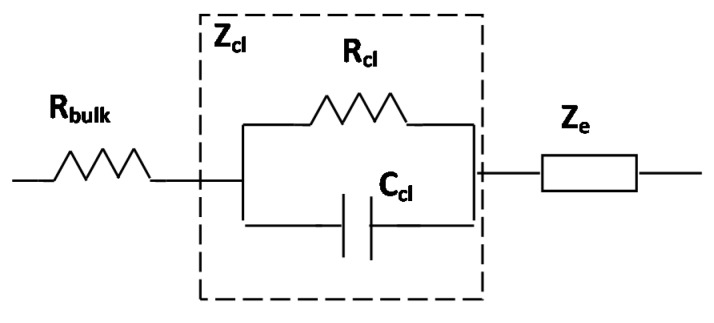
Simplified equivalent electrical circuit model of cell- substrate impedance (Z_cl_) of biological cells. C_cl_ is the capacitance of cell layer caused by dielectric properties of cell membrane, R_cl_ is the resistance across cell layer including the cell-cell resistance of the gap between cells and cell-substrate resistance. R_bulk_ is the resistance of bulk solution and the wire connection.

The name “impedance spectroscopy” is derived from the fact that the impedance is generally determined at different frequencies, rather than just one. The impedance spectroscopy measurements are generally performed using a small AC electric field over a wide frequency range (100 to 100 kHz). Thus, an impedance spectrum is obtained that allows the characterization of cell size, membrane resistance and capacitance, and cytoplasm conductivity as a function of frequency. To obtain this information, the impedance spectrum is analyzed using an equivalent electrical circuit that models the physicochemical properties of the cells biological cells. This simple electric circuit consists of resistance and capacitance connected in parallel. Figure 5 and Figure 6 illustrate the simplified equivalent electrical circuit model of cell- substrate impedance of cells. Z_cl_ is the impedance of the cell layer; it is experimentally modeled by the resistance of cell layer (R_cl_,) and the capacitance of the cell layer (C_cl_) as a parallel circuit. The cells are attached on the planar gold electrode covered with gelatine or fibronectin. The use of gelatin or fibronectin is necessary for optimization of cell adherence on electrodes. With the aid of electrical modeling of the cell-electrode system, the details of the changes in the measured impedance can be correlated to cell size, fractional electrode coverage, and cell-electrode gap. 

The measured impedance changes depend on the frequency of measurement, cell coverage, cell shapes, cell growth, and cell-electrode gap. In 1991, Giaever and Keese determined the formula for the impedance of an electrode supporting a layer of cells [84]. When a cell grows on the electrode, the cell forms focal adhesion contacts to the electrode, which accounts for 15–20% of the cell surface area. At low frequencies, current can flow from regions beneath the cell and through the medium in the cell-electrode gap, and there is no change in the measured impedance. At moderate frequencies, however, cells obstruct the current flow, and there is an increase in the measured impedance.

Determining the experimental values for the capacitance and resistance of the cell membrane will allow us to collect electrophysiological information about the cell. For instance, when the ion channels are blocked, the cell membrane will act more capacitively. The capacitance and resistance characterizing a live cell are significantly different from those of a dead cell. Healthy cells adhere more tightly to a surface in comparison to unhealthy or dead cells, which results in stronger capacitive coupling between the cells and underlying electrodes. When cells die, the impedance will change to a lower value because the membrane capacitance and resistance change.

### 3.2. Combination of ECIS Sensor with Quartz Crystal Microbalance (QCM) Resonator for Testing Water Toxicity

This review is focused on two ECIS applications that used mammalian cells BAECs cultured in vitro on the ECIS electrodes. The first application combines the ECIS technique with QCM resonator [80,81]. This combination of ECIS and QCM devices is used as a water toxicity sensor. The mammalian cells are the sensorial element, and their viability after exposure to the toxin is monitored with both sensors. The second ECIS application presented in this review is a stretchable device which has the ECIS impedance electrodes embedded in the stretchable substrate [82]. This device provides cell elongation, and at the same time, measures cell proliferation with the impedance technique.

**Figure 7:**
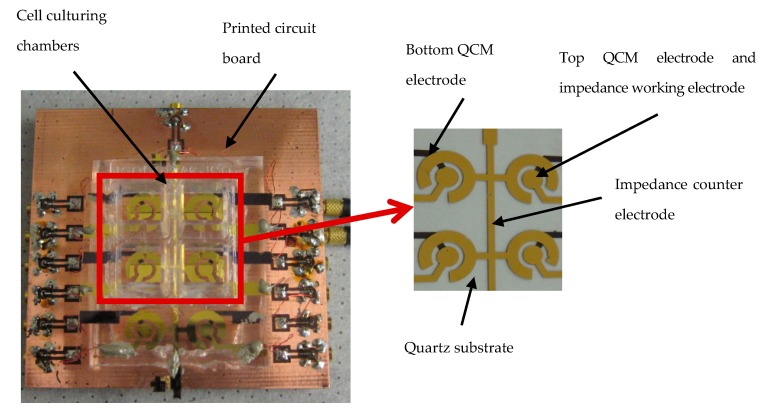
The image of the fabricated hybrid sensors configuration (2 × 3 array) on PCB with magnification of 4 sensors, adopted from [81].

**Figure 8:**
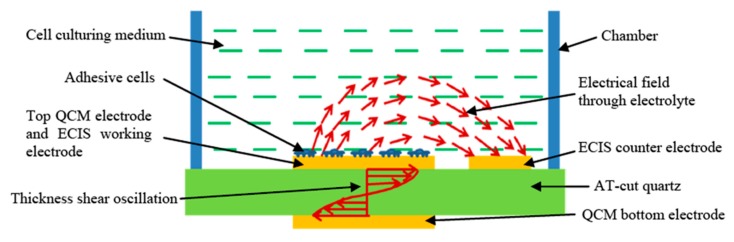
Illustration of the working principle of the hybrid biosensor which integrates the acoustic wave sensing with impedance spectroscopy technique [81].

The toxicity sensor, which is the subject of the first ECIS application of this review consisted of a combination of ECIS with QCM acoustic wave sensors fabricated on a thin, commercial, AT-cut quartz substrate. AT-cut means a way to cut the crystal, where the quartz blank is in the form of thin plate cut at an angle around 35°15′ to the optic axis of the crystal. The QCM electrodes were placed on opposite sides of the quartz substrate. The upper QCM electrode is used in an innovative way as the working electrode for the ECIS technique, as shown in Figure 7. The ECIS semicircular counter electrode was fabricated near the QCM upper electrode on the same side of the quartz substrate. The schematic of the hybrid sensor working principle is illustrated in Figure 8. An alternating current applied between the top and bottom QCM electrodes generates thickness shear-mode acoustic waves that propagate through the quartz substrate. The resonant frequency of the QCM could be calculated with Equation (1):

(1) $$f_{0}=\sqrt{\frac{\mu_{q}}{\rho_{q}}}/2t_{q}$$

where *t_q_* is the quartz crystal’s thickness, *ρ_q_* is the quartz density and *µ_q_* is the shear modulus.

Based on Equation (1), it can be seen that if the density of the QCM changes, the resonant frequency of the device also changes, making the QCM suitable for monitoring changes in mass.

In the case of this research, the mammalian cells were cultured on the combination of QCM and ECIS electrodes, which were covered with a layer of extracellular matrix (ECM) required to improve the mammalian cell attachment to the device. When the mammalian cells attached to QCM, its resonant frequency decreased. In contrast, when the mammalian cells detached from the substrate, its resonant frequency increased. When the cells were affected by drugs or toxins, they underwent apoptosis and their attachment to the QCM became less strong; eventually, the apoptotic cells detached from the QCM. Information about cell attachment and viability could be obtained by monitoring the QCM resonance frequency shifts.

The device presented in Figure 7 could simultaneously perform resonant frequency measurements and impedance measurements on the same cell monolayer cultured on the QCM upper electrode, which is also the working electrode of the ECIS system. When alternating current is applied on ECIS working and circular counter electrodes, an electrical field is generated through the cell culture medium, as seen in Figure 8. The electrical impedance between these electrodes could be recorded over a wide frequency range (40 Hz to 100 kHz) as a function of time. The amplitude of current passing through the cell is very low, in the nanoampere (nA) range. This low current creates a negligible electrical stimulation to the cell during the impedance measurement, and cell viability is not affected. The existence of membrane potential is a distinguishing feature between living and non-living cells. Impedance measurements of cells can differentiate between normal and abnormal cell types. Healthy cells adhere more tightly to a surface in comparison to unhealthy or dead cells. When cells attached and spread onto the surface of these planar electrodes for ECIS measurements, because the dielectric properties of cell membrane, the current was constrained to flow through narrow gaps between cells into the cell media, which acted as an electrolyte. Measurements of the electrical impedance of the cell-covered electrode contained information about the cell attachment, shape, and viability. Upon the attachment of cells on the electrodes, the impedance increased because the cells acted as insulating particles restricting the current flow. When the cells were apoptotic as a result of contamination or exposure to toxins or drugs, the cell impedance abruptly decreased because the cell membrane lost its dielectric properties.

The hybrid sensor was fabricated on an AT-cut quartz substrate with a nominal thickness of 100 µm, using microfabrication processes. A 20 nm chrome (Cr) layer and 200 nm of gold (Au) layer were deposited using thermal evaporation on the front side and back side of the quartz substrate. The Cr layer is necessary for increasing the adhesion of the Au layer on the quartz substrate. The circular QCM electrodes and ECIS counter electrode were patterned using photolithography and lift off techniques. The QCM top and bottom electrodes had a diameter of 2 mm. An array of six identical hybrid biosensors were fabricated on the quartz substrate, as illustrated in Figure 7. The center to center distance of the adjacent hybrid biosensors was 12 mm. This distance allows minimization of the signal interference between different channels.

This hybrid cell-based biosensor was designed to test the toxicity of water. BAECs are the primary cell type used in this toxicity device, because previous studies have determined that they are sensitive to a range of environmental toxins, and exhibit long-term survival without need for laboratory manipulation. These cells could survive for up to six weeks on the fluidic biochips, and remain responsive to toxins. The BAECs were maintained at 37 °C in MCDB-131 complete medium VEC Technology, (Greenville, PA, USA) in a 5% CO_2_ incubator.

Five different seeding densities: 500, 1.5 × 10^4^, 2.0 × 10^4^, 3.0 × 10^4^, and 5.0 × 10^4^ cells/cm^2^ were used for these experimental tests to validate the sensitivity of the device. The process of cell attachment at each seeding density was monitored repeatedly for three times and average magnitudes were taken to plot the final curve. The variations of the resonant frequency and impedance values were monitored for several hours to several days, during the entire process of cells attachment and spreading over the working electrode. 

Prior to cell inoculation, electrode arrays were cleaned by oxygen plasma for providing clean and sterilized surface for cell attachment. After plasma treatment Phosphate-Buffered Saline (PBS, 1X, GIBCO, Grand Island, NY 14072, USA) was applied to clean the electrode arrays, then 30 μg/mL fibronectin (GIBCO, Grand Island, NY 14072, USA) was coated onto the substrate of electrode arrays and keep the electrode arrays in incubator at standard cell culturing environment for 1 h before inoculating cell in the culturing chamber. After coating with fibronectin on the surface of electrode arrays, a suitable amount of BAECs cell suspension was introduced in the culturing chambers of the sensor system. The cells attach and then form a monolayer on the surface of electrodes after a certain period of time. The attachment could be verified by microscopy. Then, growth complete medium (MEM containing 5% FBS) was removed from the wells and replaced with serum-free medium, 0.5% bovine serum albumin (BSA, GIBCO, Grand Island, NY, USA) for 1~2 h to make the cell more sensitive to toxins. When toxicity testing started, the serum free medium was replaced by a different concentration of an ammonia toxin. Impedance values and resonant frequency values were monitored and recorded in real-timely for several hours after introducing ammonia toxin. The impedance curve was normalized by dividing the impedance value after introducing ammonia with the impedance values right before introducing the toxin.

**Figure 9:**
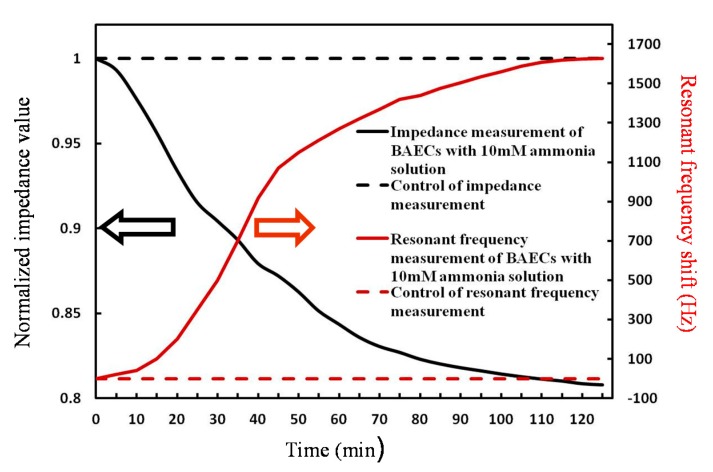
Simultaneous impedance measurements and resonant frequency measurements for testing the BAECs with the cells seeding density: 3 × 104 cells/cm^2^ with 10 mmol/L of ammonia.

When exposed to ammonia with a concentration of 10 mmol/L, the BAECs become apoptotic; see Figure 9. When the BAECs are not viable, they lose the dielectric properties of the cells membrane. The cell membrane resistance and capacitance are minimal and the final values of the impedance are equal to the value of the impedance of media solution. At the same time, the QCM resonant frequency values are increasing because the cell monolayer detach from the sensor, so the QCM becomes lighter without BAECs, and the resonant frequency values are increasing, indicating that the cells are not viable; see Figure 9. The normalized impedance values were obtained as the ratio between the measured cell impedance during the time of the experiment and the initial cell impedance. This research demonstrated the ECIS technique could be used to observe the cell reaction to toxins in water. The QCM was added as second toxicity sensor to eliminate false positives. 

### 3.3. Stretchable ECIS Sensor for Monitoring Cell Proliferation

The second ECIS device presented in this review has a stretchable substrate. This is the first time when the ECIS electrodes were embedded in stretchable and flexible device. The ECIS lab on a chip device was fabricated on a thin polydimethylsiloxane (PDMS) substrate, which can be stretched. ECIS measurements on mammalian cells exposed to cyclic strain were performed. The stretchable ECIS sensor was demonstrated to analyze the cell proliferation, determine the cell number and density, and apply mechanical stimulation.

The stretchable ECIS biosensors simulate in vitro the dynamic environment of organism, such as pulsation, bending, and stretching, which enables investigations on cell behavior that undergoes mechanical stimuli in biological tissue. BAECs have been used in this research, because they are exposed in vivo to cyclic physiologic elongation produced by the blood circulation in the arteries. The stretchable ECIS biosensor was used to analyze the proliferation of BAECs under different cyclic mechanical stimulation.

**Figure 10:**
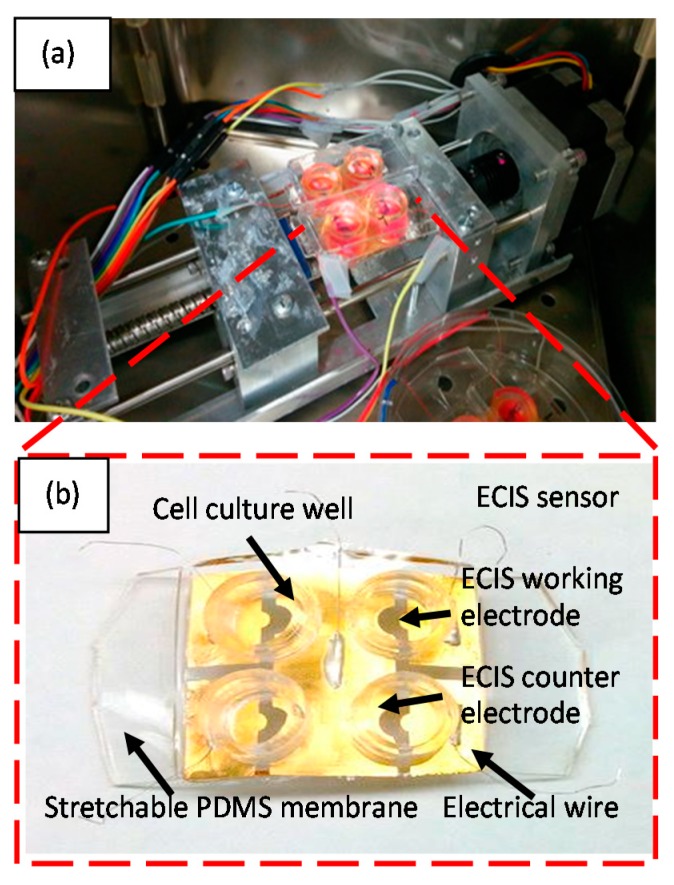
(**a**) A linear motor was used to cyclically stretch the stretchable ECIS sensor. The linear motor with the stretchable ECIS sensor mounted on it was placed inside the incubator; (**b**) Stretchable ECIS sensor. The stretchable ECIS sensors were connected to impedance analyser 4294A, which applied alternating current (AC) between working and counter electrodes and record the impedance data, copied with permission from [82].

The experimental setup is shown in Figure 10. A linear slide system (Haydon Kerk, Waterbury, CT, USA) (Figure 10a) was used to cyclically stretch the ECIS sensor array (Figure 10b). As illustrated in Figure 10a, the sensor array contains four ECIS sensors, with a cell culture well fabricated on top of each ECIS sensor. The linear slide system with the stretchable ECIS sensor array mounted on it, was kept in a cell culture incubator during the experiment. The four ECIS sensors included four individual working electrodes and one common counter electrode were connected to an Impedance Analyzer (type 4294A, Agilent, Santa Clara, CA 95051, USA) with Multiplexer (type DG408, Maxim Integrated Products, Sunnyvale, CA 94086, USA). During the experiment, the electrical pulses were sent to a computer controlled by Labview program (version 2015). The impedance data was acquired at the end of each stretch/release cycle. 

The stretchable substrate of the sensor was fabricated from PDMS (Sylgard 184 silicone elastomer kit, ML SOLAR, Campbell, CA, USA). The PDMS precursor was mixed with the crosslinking agent with a weight ratio of 10:1. Initially Au electrodes were fabricated on a glass slide, and later, the Au electrodes were transferred to the PDMS substrate. A gold film with the thickness of 50 nm was deposited using sputtering equipment (Hummer XP, Anatech, Union City, CA, USA) on a clean glass slide pre-covered with a metal shadow mask. The weak adhesion between gold and glass allowed the gold film later to detach from glass slide. The glass slide with the gold electrodes covered with silane and uncured PDMS was stamped onto pre-stretched 20% PDMS substrate. After curing the PDMS at 150 °C for 24 h, the glass slide was peeled off from the PDMS substrate. The gold pattern adhered on the pre-stretched PDMS substrate.

An important aspect of the stretchable device is to cover for protection the ECIS electrodes with a thin PDMS film. This upper PDMS layer impeded the breaking and buckling of gold electrodes during cyclic stretching and releasing. In order to obtain a thin protection film, PDMS diluted with hexane was spin-coated (2000 rpm) on the gold pattern. Openings of 400 µm diameters were fabricated in the top PDMS protection film, since for performing ECIS measurements, the Au film has to be in direct contact with the mammalian cells under study. The ECIS electrodes consisted of a circular working electrode and a semi -circular counter electrode. Inductive coupled plasma (ICP) was used to etch the top protective layer of PDMS, which was pre-covered with a shadow mask. The dimensions of working electrodes were 400 µm diameter, which was defined by the shadow mask. The measured electrical impedance is mainly determined by the working electrode with very small surface area [80]. The area of counter electrode needs to be large enough to provide sufficient current paths and circuit connection.

**Figure 11:**
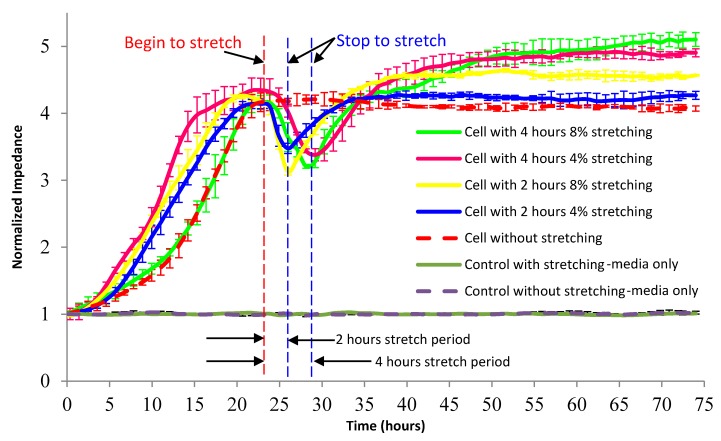
Normalized impedance response of cells and cell culture medium from the stretchable impedance sensors (*n* = 3). The 4% or 8% stretch with 1 Hz was only applied from 24 h to 26 h or from 24 h to 28 h. The controls only contained cell culture medium, copied with permission from [82].

The ECIS technique was used in this research to monitor the impedance of BAECs cell membrane. In order to decide the best frequency value for performing cell membrane impedance measurements, initially the impedance values were recorded over a large frequency range. These impedance measurements were performed from 40 Hz to 100 kHz, between the ECIS working and counter electrodes, using the impedance analyzer. Therefore, 9 kHz was used as optimal frequency for all the impedance measurements presented in this paper. The normalized impedance response of cells and cell culture medium from the stretchable ECIS sensors (n = 3) is shown in Figure 11. The normalized impedance values were obtained as the ratio between the measured cell impedance during the time of the experiment and the initial cell impedance. Each experiment was repeated three times, n = 3. BAECs cells were cultured on the ECIS electrodes. 

Figure 11 shows 4% or 8% stretch with the cyclic frequency of 1 Hz applied for 2 or 4 h. The frequency for stretching cycles was 1 Hz to simulate the adult heartbeat. The controls only contained cell culture medium. The solid green, pink, yellow, and blue lines represented the normalized impedance of cell with stretching respectively either from 24 h to 26 h or from 24 h to 28 h. The dashed red lines represented the normalized impedance of cell without stretching. The light green line represents the normalized impedance of cell culture medium with 8% stretching from 24 h to 28 h. The dashed purple lines represent the normalized impedance of cell culture medium without stretching. 

In Figure 11, the normalized cell impedance increased gradually after cell seeding, and then reached stable values for the cells without stretching, similar to the impedance response in previous research [82]. All the values of normalized cell impedance did not fluctuate dramatically or shift to extremely high levels during the experiment. The impedance of control with or without stretch is overlapped in most of the area during the cyclic stretching, which indicates the stretchable ECIS sensors can keep good conductive ability during cyclic stretching, because the gold electrodes were fabricated on the substrate with 20% pre-stretched. The impedance measurements were carried out when the sensors were in un-stretch status. Even though there are cracks on the gold electrodes, 20% pre-stretching allow the electrodes to keep good conductive ability in an un-stretched state. 

It was most likely that the stretch on cells influence the cell-substrate attachment and/or cell-cell contacts, and higher stretch magnitude further influence the attachment and/or junction. The stretching stopped at 26 h or 28 h, and the normalized impedance gradually increased to the level before stretch because of the recovery of cell-substrate attachment and cell-cell contacts. Finally, the normalized impedance of confluent cells with different stretch stimuli increased to a higher level than that without stretching, as shown in Figure 11. Perhaps one of the reasons was the cell proliferation, and higher impedance slope indicated higher proliferation rate. This application of stretchable ECIS technique demonstrated that when the BAECs are exposed to cyclic elongation for a period of time the cell proliferation is more intens in comparision with the situation when the cells are cultured without applied elongation. 

## 4. Conclusions

This paper presents MEMS and nanotechnology biosensors researched and developed by the authors of this review. The presented sensors with dimensions at the nano- and micro-scale were used for biological applications focused on live cells. The biosensors in this review are: the cantilever beam and electrochemical sensor based on electric cell-substrate impedance sensing (ECIS) technique. The cantilever beams presented in this review are used as temperature sensors and are operated in deflection mode or in resonant frequency mode. The ECIS technique presented in this review was used to study the mammalian cells viability. 

The methods for temperature measurements with cantilever beam were based on bimaterial cantilever beam deflection and also monitoring the cantilever beam vibrations. The temperature variations of single cells are very small, and both types of cantilever beams were demonstrated to detect temperature variations produced by single BFC when activated with NE. 

The ECIS technique combined with QCM was used for water toxicity studies. Cell damage induced by toxic water resulted in a decrease in impedance, as well as an increase in the resonant frequency. The effects of the toxins—ammonia, nicotine, and aldicarb—on cells were monitored with both sensors using the QCM and the ECIS technique. The lab on chip was demonstrated to indicate low concentration of toxins. The responses of BAECs to toxic samples occurred during initial 5 to 20 min depending on the type of chemicals and concentrations. A highly linear correlation between signal shifts and chemical concentrations was demonstrated for each toxin. This review also presented ECIS electrodes fabricated on a stretchable substrate. ECIS measurements on mammalian cells exposed to cyclic strain of 4% and 8% were successfully demonstrated. Bovine aortic endothelial cells (BAEC) were used to evaluate the endothelial function influenced by mechanical stimuli in this research because they undergo in vivo cyclic physiologic elongation produced by the blood circulation in the arteries. Using impedance measurements, it was demonstrated that the cyclic stretch improved cell attachment and proliferation.

The cantilever beam sensors or ECIS technique are very different, as physical concepts or mode of operations but their fabrication is based on the classic microfabrication techniques.
